# Esculetin Downregulates the Expression of AML1-ETO and C-Kit in Kasumi-1 Cell Line by Decreasing Half-Life of mRNA

**DOI:** 10.1155/2015/781473

**Published:** 2015-03-11

**Authors:** Sharad Sawney, Rashi Arora, Kamal K. Aggarwal, Daman Saluja

**Affiliations:** ^1^University School of Biotechnology, Guru Gobind Singh Indraprastha University, Sector 16-C, Dwarka, New Delhi 110078, India; ^2^Dr. B.R. Ambedkar Center for Biomedical Research University of Delhi, Delhi 110007, India

## Abstract

One of the most frequent genetic aberrations in acute myeloid leukemia (AML) is chromosomal translocation between *AML1/RUNX1* on chromosome 21 and *ETO* gene on chromosome 8 resulting in the expression of chimeric oncogene *AML1-ETO*. Although patients with t(8;21) translocation have good prognosis, 5-year survival is observed only in 50% of the cases. AML1-ETO translocation is usually accompanied by overexpression of mutant *C-Kit*, a tyrosine kinase, which contributes to uncontrolled proliferation of premature blood cells leading to relapse and poor prognosis. We illustrate the potential use of esculetin on leukemic cell line, Kasumi-1, bearing t(8;21) translocation and mutated *C-Kit* gene. Esculetin decreases the expression of AML1-ETO at both protein and transcript level within 24 hours of treatment. Half-life of AML1-ETO mRNA was reduced from 7 hours to 1.5 hours. Similarly half-life of C-Kit mRNA was reduced to 2 hours from 5 hours in esculetin treated cells. Esculetin also perturbed the expression of ectopically expressed AML1-ETO in U937 cells. The decreased expression of* AML1-ETO* chimeric gene was associated with increased expression of *LAT1* and *RUNX3* genes, targets of AML1. We envisage that discovery of a drug candidate which could target both these mutated genes would be a considerable breakthrough for future application.

## 1. Introduction

The World Health Organization reported 14.1 million new cases and 8.2 million cancer deaths worldwide in 2012. It is prognosticated that, over the next few years, cancer including leukemia will surpass cardiovascular diseases [1]. Based on the clinical onset and lineage of the transformed immature leukocytes, leukemia can be acute or chronic and myeloid or lymphoid in origin. Chromosomal abnormalities, translocations, gene mutations, deregulation of gene expression, and epigenetic transmutations in the hematopoietic stem cells are the major contributory factors that interfere with the hematopoietic differentiation and result in the development of leukemia. Acute myeloid leukemia (AML) is a major class of leukemia, accounting for 20% of acute leukemia, wherein role of translocations and mutations in the malignancy of hematopoietic system is well established [2]. These genetic alterations broadly fall in two categories, Class I and Class II.

Class I is comprised of mutations in genes such as tyrosine kinases (*KIT* and* FLT3* genes) that alter signal transduction pathways and thereby increase cellular proliferation and/or survival of hematopoietic progenitor cells [3]. Mutations and alterations in transcription factors, such as AML1 (RUNX1), CEBPA, and MLL, resulting in the disruption of cellular differentiation come under Class II mutation. Class II mutations, though the first hit, are insufficient to cause leukemia and require secondary hit in genes of Class I for the phenotype [4]. Most prevalent Class II mutations under AML are translocation between* RUNX1* (*CBFα*) gene on chromosome 21,* ETO* (or* MTG8*) gene on the 8 chromosome t(8;21) (q22;q22) [5], and inversion inv16 (*CBFβ-MYH11*) (p13q22) [6]. The t(8;21) results in a chimeric oncogene* AML1-ETO*, resulting in expression of a fused oncoprotein AML1-ETO (AE), and is found in 20% of the AML patients [2, 7]. The conserved Runt Homology Domain (RHD) required for DNA binding is at the N terminal of AML1 protein, whereas the transactivation and repressor domains span the C terminal region of the protein [1].* AML1* gene acts as a transcription activator for majority of hematopoietic genes like T and B-cell receptors,* PU1*,* GATA*, interleukin-3 (*IL-3*), macrophage colony stimulating factor receptor (*M-CSFR*), and so forth [8]. The ETO protein, a corepressor, is characterized by its four conserved Nervy Homology Regions (NHR1 to NHR4) that play an important role in protein-protein interactions [9]. The chimeric AML1-ETO protein is comprised of N terminal DNA binding domain of* AML1* while the* AML1* activation domains are replaced with almost the entire ETO protein. As a result, the AE chimeric protein inhibits the expression of* AML1* target genes instead of activating them through recruitment of corepressors and HDACs [10]. Interactions within the family of ETO proteins result in the formation of high molecular weight oligomers, further increasing the association of AE protein with N-CoR, SMRT, and other corepressors. These interactions are indispensable for the functioning of the chimeric AML1-ETO oncoprotein [9, 11].

The AML1-ETO repressor protein actively competes with AML1 and suppresses the transcription of* AML1* target genes such as E-proteins, *PU*.1,* C/EBP*α*,* and* GATA-1* transcription factors that play a vital role in hematopoiesis [2, 10]. Genes of DNA repair pathway are also inhibited by AE protein, resulting in the uninterrupted expansion of the mutated diseased cells [12].

The mutations in* AML1* mark the genesis of leukemia; however, accumulation of secondary mutations are necessary for the manifestation of aggressive forms of acute leukemia [13]. About 70% of AML cases witness overexpression of receptor tyrosine kinase KIT. Though mutations in* KIT* gene are sporadic (as low as ~5%-6%) [14, 15], its incidence increases to as high as 30% in patients (37% of adult cases and 19% of paediatric cases) with AE translocation and is associated with poor prognosis [3, 16].

The current treatment of AML1-ETO acute myeloid leukemia involves the use of cytarabine (Ara-C) and anthracycline (such as daunorubicin). These drugs often result in complete remission in 60 to 80% of patients with a median survival less than 2 years and an overall survival of about 5 years [17]. Therefore, identification of novel therapeutic targets and drugs for treatment of t(8;21) positive AML is necessary for better patient management. The discovery of imatinib (Gleevec), an inhibitor of tyrosine kinase activity of BCR/ABL, was a breakthrough in the treatment of chronic myeloid leukemia (CML). It has the potential to block the activity of mutated* KIT* and* PDGFR* and has proven to be a promising candidate for the treatment of CML. Along similar lines, attempts are being made to modulate the levels of AE oncoprotein in t(8;21) positive AML patients. Calpain B inhibitors have been shown to trigger AE protein degradation and decrease cell viability and clonogenic potential in Kasumi-1 cells [18].

The ectopic expression of Cathepsin G, a serine protease, induces degradation of AE protein in Kasumi-1 cells [19]. Similarly, corticosteroids were also shown to trigger ubiquitin mediated proteasomal degradation of AE chimeric protein [20]. Exploring the effect of natural compounds from traditionally known medicinal plants has attracted increasing attention in recent years. Oridonin, a diterpenoid, isolated from* Isodon rubescens*, was shown to cleave AML1-ETO protein in caspase-3 dependent manner [21].

Esculetin, a natural coumarin found in several plants including* Artemisia capillaris,* leaves of* Citrus limonia* [22], and* Ceratostigma willmottianum* [23], has been shown to protect against N-methyl-N-nitrosourea-induced mammary carcinogenesis in rodents [24]. It inhibits proliferation of human breast cancer cells [25] and induces apoptosis in human leukemic HL-60 cell line [26]. Esculetin is, therefore, magnetizing the focus of active research as a potential anticancer drug. We tested whether esculetin can categorically target AML1-ETO so that it could be used in treating t(8;21) AML. Since C-Kit mutations are highly prevalent in AML1-ETO patients (about 30% cases), we also compared the levels of C-Kit in the presence and absence of esculetin using Kasumi-1 cell line as it possesses both AML1-ETO and C-Kit activating mutation N822K. Our results illustrate that esculetin has the antiproliferating activity against Kasumi-1 cell line. Our results evince that esculetin decreased the levels of both C-Kit protein and AML1-ETO chimeric protein. Furthermore, the expression of both the genes is reduced drastically at the transcript level due to enhanced degradation of the AML1-ETO and C-Kit mRNA.

## 2. Materials and Methods 

### 2.1. Materials

Esculetin (6,7-dihydroxycoumarin, 98% purity) was purchased from Sigma-Aldrich and dissolved in dimethyl sulfoxide (DMSO, vehicle). RPMI 1640 and all other chemicals, not specifically cited here, were purchased from Sigma-Aldrich. Fetal bovine serum (FBS) was purchased from GIBCO-BRL (South America Origin). An enhanced chemiluminescence (ECL) kit was purchased from Pierce. Antibody against AML1-ETO chimeric protein was purchased from Diagenode (C15410080) (Belgium). Antibody against C-Kit and *β*-actin was purchased from Cell Signaling (USA). Peroxidase-labeled donkey anti-rabbit and anti-mouse immunoglobulin was purchased from Santa Cruz (USA).

### 2.2. Leukemia Cell Lines

Kasumi-1, a human leukemia cell line (M2 AML), was a kind gift from Professor Olef Hendrich (Newcastle University, Northern Institute for Cancer Research, UK). U937 and U937AE cell lines were a kind gift from Professor Dong-Er Zhang (Department of Pathology & Division of Biological Sciences Moores, UCSD Cancer Center, University of California, San Diego, USA). Kasumi-1 and U937 were grown in RPMI-1640 (Sigma) enriched with 15% fetal bovine serum (Gibco, South American Origin) at 37°C under 5% CO_2_ atmosphere. U937 and U937AE were maintained as described by Burel et al. [27]. Culture media were changed periodically to ensure the cellular integrity.

### 2.3. RNA Isolation and Real Time PCR

For gene expression studies at mRNA level, Kasumi-1 cells were treated with 100 *μ*M esculetin for 24 hours and 48 hours. To determine the half-life of AML1-ETO and C-Kit mRNA, Kasumi-1 cells treated with 100 *μ*M esculetin for 24 hours were subsequently treated with 50 *μ*M of *α*-amanitin for different time intervals [28]. A control set without esculetin treatment was analysed in parallel. The expression of mRNA of AML1-ETO was also compared in U937AE cell line by culturing the cells in absence of tetracycline for 24 hours, to induce transcription of* AML1-ETO*. Thereafter cells were incubated with or without esculetin for 48 hours. Total RNA was isolated using RNA easy kit (Qiagen) as per manufacturer's protocol. RNA concentration was determined using Nanodrop (Thermo) and the quality and integrity were checked on 1% agarose gel. First strand cDNA was synthesized using 1 *μ*g of total RNA in 20 *μ*L reverse transcriptase reaction mixture according to the manufacturer's protocol (Thermo). Semiquantitative PCR was carried out typically in 20 *μ*L PCR reaction mixture using primers specific for* AML1-ETO* and* C-Kit* (5 pmol each) and 1/10 volume of cDNA preparation (1 *μ*L) as template, PCR buffer with MgCl_2,_ 2 mM dNTPs, (final concentration 0.2 mM) and Taq polymerase (1 unit). Initial denaturation was performed at 95°C followed by 28 cycles of denaturation at 95°C for 30 seconds, annealing at 60°C for 30 sec, and extension at 72°C for 30 sec. Amplicons were electrophoresed on 1-2% agarose gel. For quantitative PCR, 1X SYBR Green dye mix (Roche), 2 pmoles of each primer, and 1/20 volume of cDNA was used in 20 *μ*L reaction on the BIO-RAD iCycler iQ system (BioRad, Hercules, CA, USA). The fluorescence threshold value was calculated using the iCycle iQ system software. Copy number for a given RNA was calculated using standard curve of known template concentration. Standard curve was made by plotting fluorescence obtained in qPCR against the serially diluted known concentration of plasmid having AML1-ETO and C-Kit DNA fragments used for qPCR.* 18S rRNAs* and* GUSB* were taken as internal control [29] (see Table 1).

### 2.4. Preparation of Total Cell Extracts and Immunoblot Analysis

After the desired period of incubation with esculetin (100 *μ*M for 12–48 hours), cells were washed with PBS and resuspended in RIPA lysis buffer. The mixture was centrifuged for 15 min (10,000 g) after mixing for 30 min at 4°C. Protein concentration in the supernatant (whole cell lysate) was determined using BCA protein estimation kit (Bangalore Genei) and equal amount of protein of control and esculetin treated sample (100 *μ*M) was resolved on 8% SDS-PAGE and transferred onto PVDF membrane. The Western blot was probed for the desired proteins and detected using ECL kit (Pierce) followed by exposure on luminescent image analyzer (Fujifilm LAS-4000).

### 2.5. Statistical Analysis

Unless otherwise indicated, q-PCR and Western blot analysis were repeated at least thrice. The results of various parameters were analysed using Graph Pad Prism 6.0. All the data was expressed as mean ± SD. The statistical significance was assessed by one way and two way analyses of variance followed by Bonferroni's multiple comparisons test with a confidence level of (*P* < 0.05).

## 3. Results and Discussion

Progression of acute leukemia is associated with the partial loss in the function of homeostasis of hematopoietic stem cells [30]. Acute myeloid leukemia patients harbouring the t(8;21) translocation are generally given a good prognosis at the initiation of treatment and absolute remission is achieved in most of the cases. Dismally, the disease recurs in 30–40% of these patients with overall survival being 5 years in less than 50% of the patients [31]. Higher relapse rate and shorter life span are witnessed in cases with high expression of* C-Kit* mutations [32]. As in the case of U937 and HL-60 cell lines [26, 33], we observed concentration and time dependent antiproliferating activity of esculetin against Kasumi-1 cell line, which possess both AML1-ETO t(8 : 21) and C-Kit activating mutation N822K (unpublished data). Translocations and mutations compliment the proliferation activity, progression of malignancy, and aggressiveness of the cancer. Multiple mutations in* C-Kit* gene along with* AML1-ETO* oncogene are the most common pair of genetic anomalies found in t(8;21) type of AML. Thus, there is an exigency to identify a molecule which can simultaneously target both these genes for the efficacious treatment and preponderant outcomes [1, 34].

### 3.1. Effect of Esculetin on Expression of Chimeric Proteins

**Figure 1:**
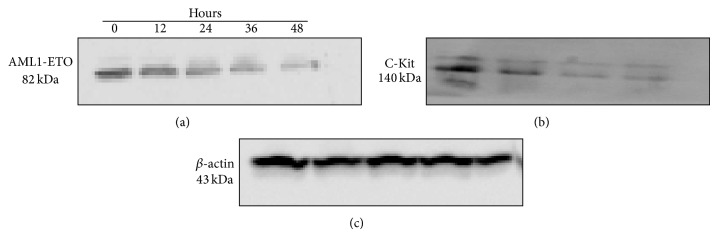
Esculetin treatment downregulates the expression of AML1-ETO chimeric protein and C-Kit protein in Kausmi-1 cell line. Whole cell lysates from Kausmi-1 cells treated with 100 *μ*M of esculetin for the indicated periods of time (in hours) were analysed. Equal amount of protein is loaded in each lane of the denaturing gel and immunoblotted with anti-AML1-ETO (a), anti-C-Kit (b), and anti-*β*-actin antibodies (c); *β*-actin was used as the loading control.

Oridonin, a tetracycline diterpenoid compound, was recently shown to degrade the chimeric AML1-ETO protein in a caspase-3 dependent manner, although oridonin has no effect on the transcription level of AML1-ETO [21]. We, therefore, examined the effect of esculetin on the expression of not only chimeric AML1-ETO protein but also C-Kit, a tyrosine kinase, using Kasumi-1 cell line as a model system. Kasumi-1 cells were treated with 100 *μ*M esculetin along with controls and protein was resolved on 8% SDS PAGE and probed with anti-AE and anti-C-Kit antibody. As shown in Figure 1(a), the expression of AE protein decreased as a function of time of esculetin treatment. Interestingly, we also observed a parallel decrease in the expression of C-Kit in esculetin treated Kasumi-1 cells (Figure 1(b)). Importantly, esculetin treatment of cells did not result in the generation of cleaved form (delta form) of AML1-ETO chimeric protein. This is in contrast to the observations reported earlier wherein oridonin treatment of Kasumi-1 cell line was shown to cleave the chimeric AML1-ETO protein [21]. This impelled us to examine whether this decrease in the level of protein is due to modulation of transcription of these genes.

### 3.2. Effect of Esculetin on Expression of Chimeric Gene

**Figure 2:**
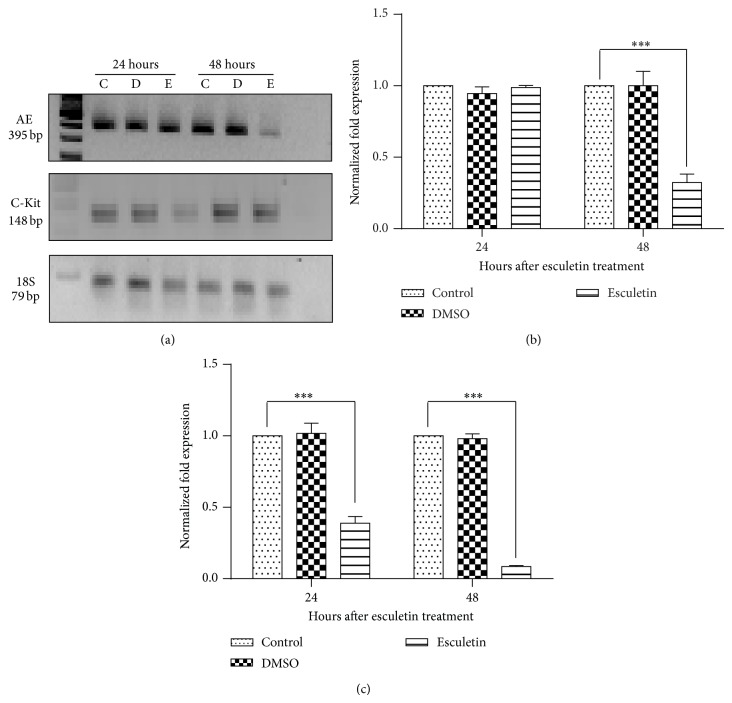
Esculetin treatment alters the transcript levels of AML1-ETO and C-Kit in Kausmi-1 cells. Kausmi-1 cells were used in these analyses. The results of semiquantitative PCR were for AML1-ETO and C-Kit using cDNA as template; Kausmi-1 cells were incubated with 100 *μ*M esculetin (E) and DMSO (D) (vehicle control) along with control untreated (C) for 24 hours and 48 hours (a); results of qPCR for AML1-ETO (b) and C-Kit (c) in esculetin treated and control Kausmi-1 cells are shown. The duration of treatment is indicated on top of the lanes (I).* 18SrRNA* was used as an internal control for semiquantitative PCR; for qPCR* 18SrRNA* and* GUSB* were used for normalization. The data represents mean ± SD. ^***^ *P* < 0.05 compared to control.

**Figure 3:**
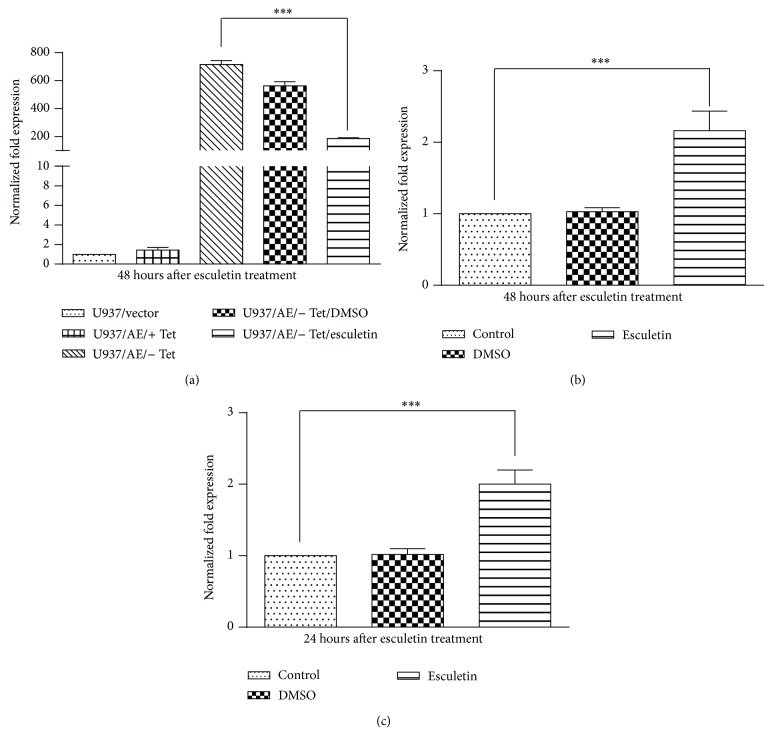
Effect of esculetin on the expression of AML1-ETO in transfected U937 cells (a). AML1-ETO expression was analyzed in untreated U937 cells/vector, U937/AE/+ Tet/, U937/AE/− Tet/, U937/AE/− Tet/D, and U937/AE/− Tet/E 48 hours after esculetin treatment. AML1-ETO (AE), tetracycline (Tet), DMSO (D), and esculetin (E). Esculetin affects the expression of* LAT1* and* RUNX3* genes, targets of AML1 (a, b, and c). Kasumi-1 cells were treated with 100 *μ*M of esculetin for 48 hours for* LAT1* and 24 hours for* RUNX3*; mRNA expression level for both the genes was determined using quantitative real time PCR.* 18SrRNA* and* GUSB* genes were used for normalization. An increase in the expression of* LAT1* and* RUNX3* gene suggests increased expression of AML-1 targets. The data represents mean ± SD, ^***^ *P* < 0.05 compared to control.

To examine the effect of esculetin on transcription of* AML1-ETO* and* C-Kit* gene, Kasumi-1 cells were treated with 100 *μ*M of esculetin, RNA was isolated, and expression was analysed by semiquantitative RT-PCR. As shown in Figure 2(a), esculetin treatment for 48 hours downregulates the expression of* AML1-ETO* gene as compared to the DMSO vehicle control. Importantly, the expression of mutated* C-Kit* gene was also found to be substantially downregulated at 24 hours of esculetin treatment and it reached below the detectable limits by 48 hours. This observation was further substantiated by quantitative RT-PCR analysis, wherein the expression of* AML1-ETO* and* C-Kit* was compared in Kasumi-1 cells after 24 and 48 hours of esculetin treatment to untreated controls (Figures 2(b) and 2(c)).* C-Kit* expression was downregulated more than 2-fold within 24 hours of esculetin treatment, while at 48 hours, the expression was almost imperceptible. Similarly, esculetin resulted in about 60% inhibition of* AML1-ETO* expression at 48 hours.* 18S rRNA* and* glucuronidase beta (GUSB)* gene expression was used to normalize the data. These observations suggested that esculetin mediated decrease in the protein expression of AE as well as C-Kit could possibly be due to decreased transcript rather than the cleavage of the protein. We also compared the expression of chimeric gene in U937AE cells stably transfected with recombinant plasmid carrying AML1-ETO c-DNA (please see Section 2 for details). Esculetin treatment substantially downregulated the levels of ectopically expressed* AML1-ETO* (Figure 3(a)). Since the chimeric gene in these cells was not under the regulation of natural promoter, this observation also suggested that esculetin may not be affecting the transcription initiation; rather its effect may be posttranscriptional. Further, we observed that the decrease in* AML1-ETO* levels in Kasumi-1 cells treated with esculetin was accompanied by an increase in the expression of AML-1 target genes, namely,* LAT1* (Figure 3(b)) and* RUNX3* (Figure 3(c)). The level of RUNX-1, mRNA was however not significantly altered in the presence of esculetin. Based on these observations, we propose that, under reduced levels of the chimeric protein, which acts as a repressor, the normal RUNX-1 protein is now able to activate the expression of its target genes. These results also suggested that the effect of esculetin on C-Kit and AML1-ETO transcript is specific and not due to general transcription inhibition and/or degradation of mRNA.

### 3.3. Effect of Esculetin on Half-Life of Chimeric Transcript

**Figure 4:**
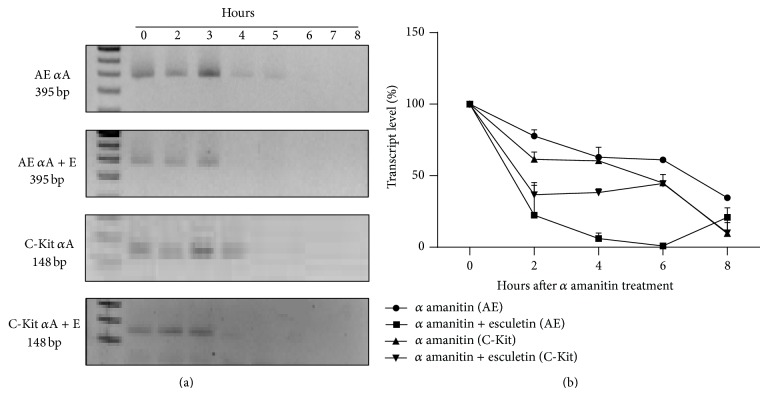
Esculetin treatment reduces half-life of AML1-ETO and C-Kit mRNA. The levels of mRNA for AML1-ETO (AE) and C-Kit were quantified by semiquantitative RT-PCR (a) and qPCR (b) in esculetin (E) treated and untreated Kausmi-1 cells incubated with *α* amanitin (*α*A) for 2–8 hours.

To further analyse if this decrease in the transcript level is due to decrease in transcription or due to increased degradation, we determined the half-life of the mRNA for C-Kit and AML1-ETO. Transcription of mRNA was inhibited in esculetin treated and untreated Kasumi-1 cells by incubating the Kasumi-1 cells in medium containing 50 *μ*M of *α*-amanitin, an intense inhibitor of RNA polymerase II [35]. Total RNA was isolated from these cells at defined time interims and subjected to qPCR as described in Section 2. RNA concentration was estimated utilizing standard graph generated from cloned cDNA of AML1-ETO and C-Kit as described in Section 2. Interestingly, we observed a substantial decrease in the half-life of mRNA both for AML1-ETO and C-Kit (see Figure 4(a)). In the absence of esculetin, the half-life of AML1-ETO mRNA was determined to be 7 hours, while it diminished to 1.5 hours in the presence of esculetin. Similarly, the half-life of C-Kit mRNA was reduced from 5 hours in untreated control cells to 2 hours when cells were treated with esculetin (see Figure 4(b)).

## 4. Conclusion 

Our experiments clearly establish that esculetin reduces the levels of* C-Kit* as well as chimeric gene* AML1-ETO* by altering the half-life of mRNA. Although, at present the mechanism by which esculetin reduces the half-life of mRNA is not known, future studies are being directed to check the effect of esculetin in posttranscriptional modifications and/or splicing of precursor RNA. Earlier, the siRNA mediated “knock-down” of the mRNA of AML-ETO was shown to result in growth arrest, granulocytic differentiation, and modulation of expression of genes involved in cell-cycle control and differentiation [36, 37]. To the best of our knowledge, this is the first report wherein a natural coumarin has been shown to regulate the expression of oncogenes, namely,* AML1-ETO* and* C-Kit*, at the posttranscriptional level. It appears that the impact of esculetin on the levels of both AML1-ETO and C-Kit may be a consequence of an indirect mechanism. It is possible that such a mechanism may involve the downregulation of another transcription factor. We, therefore, propose that use of esculetin may provide an important therapeutic approach for t(8;21) positive leukemia.

## Figures and Tables

**Table 1:** 

Primer name	Forward primers	Reverse primer	Product size (bp)
*GUSB *	CTCATTTGGAATTTTGCCGATT	CCGAGTGAAGATCCCCTTTTTA	80
*18S rRNAS *	GTGGTGTTGAGGAAAGCAGACA	TGATCACACGTTCCACCTCATC	79
*AML1-ETO *	CTACCGCAGCCATGAAGAACC	AGAGGAAGGCCCATTGCTGAA	395
*C-Kit *	CACCGAAGGAGGCACTTACAC	GGAATCCTGCTGCCACACA	148
*LAT1 *	GAGCTACGAGAACGAGGGTG	GCCTGGGTTGTGATAGTCGT	157
*RUNX3 *	GGATGGTACGGTGGTGACTG	CTTGATGGCTCGGTGGTAGG	208
